# American Investigation in Turkey

**Published:** 1881-09

**Authors:** 


### AMERICAN INVESTIGATIONS IN TURKEY.

Consul-General Heap, of Constantinople, in a recent dispatch, informs the State Department at Washington that the Turkish authories have determined to grant the concession desired by Professor Charles Eliot Norton of Harvard University, of the right to investigate the remains of an ancient Greek city upon Turkish soil. It will take some weeks for the concession to go through all the red tape process of the Turkish Government, but there is no reason to doubt that the expedition will be on its way next month, and that the work will begin at the ancient city early in February. This expedition is unprecedented.

